# Actigraphic measurement of the upper limbs movements in acute stroke patients

**DOI:** 10.1186/s12984-019-0603-z

**Published:** 2019-12-04

**Authors:** Chiara Iacovelli, Pietro Caliandro, Marco Rabuffetti, Luca Padua, Chiara Simbolotti, Giuseppe Reale, Maurizio Ferrarin, Paolo Maria Rossini

**Affiliations:** 1 IRCCS Fondazione Don Carlo Gnocchi, Milan, Italy; 2 Complex Operative Unit of Neurology, Fondazione Policlinico Universitario A. Gemelli IRCSS, L.go F. Vito, 1, 00168 Rome, Italy; 3 Department of Geriatrics Neurosciences and Orthopaedics, Università Cattolica del Sacro Cuore, Rome, Italy; 4 Institute of Neurology, Department Geriatrics, Neuroscience & Orthopedics, Catholic University, Policlinic A. Gemelli, Rome, Italy

**Keywords:** Actigraphy, Disability motor evaluation, Multiparametric monitoring, Stroke, Innovative biotechnologies

## Abstract

**Background:**

Stroke units provide patients with a multiparametric monitoring of vital functions, while no instruments are actually available for a continuous monitoring of patients motor performance. Our aim was to develop an actigraphic index able both to identify the paretic limb and continuously monitor the motor performance of stroke patients in the stroke unit environment.

**Methods:**

Twenty consecutive acute stroke patients (mean age 69.2 years SD 10.1, 8 males and 12 females) and 17 bed-restrained patients (mean age 70.5 years SD 7.3, 7 males and 10 females) hospitalized for orthopedic diseases of the lower limbs, but not experiencing neurological symptoms, were enrolled. This last group represented our control group. The motor activity of arms was recorded for 24 h using two programmable actigraphic systems showing off as wrist-worn watches. The firmware segmented the acquisition in epochs of 1 minute and for each epoch calculates two motor activity indices: MA_e1_ (Epoch-related Motor Activity index) and MA_e2_ (Epoch-related Motor Activity index 2). MA_e1_ is defined as the standard deviation of the acceleration module and MA_e2_ as the module of the standard deviation of acceleration components. To describe the 24 h motor performance of each limb, we calculated the mean value of MA_e1_ and MA_e2_ (respectively MA_1_24h_ and MA_2_24h_). Then we obtained two Asymmetry Rate Indices: AR_1_24h_ and AR_2_24h_ to show the motor activity prevalence. AR_1_24h_ refers to the asymmetry index between the values of MA_e1_ of both arms and AR_2_24h_ to MA_e2_ values.

The stroke patients were clinically evaluated by NIHSS at the beginning (NIHSS_T0_) and at the end (NIHSS_T1_) of the 24 h actigraphic recordings.

**Results:**

Both MA_1_24h_ and MA_2_24h_ indices were smaller in the paretic than in the unaffected arm (respectively *p* = 0.004 and *p* = 0.004). AR_2_24h_ showed a better capability (95% of paretic arms correctly identified, Phi Coefficient: 0.903) to discriminate the laterality of the clinical deficit than AR_1_24h_ (85% of paretic arms correctly identified, Phi Coefficient: 0,698). We also found that AR_1_24h_ did not differ between the two groups of patients while AR_2_24h_ was greater in stroke patients than in controls and positively correlated with NIHSS total scores (r: 0.714, *p* < 0.001 for NIHSS, IC95%: 0.42–0.90) and with the sub-score relative to the paretic upper limb (r: 0.812, *p* < 0.001, IC95%: 0.62–0.96).

**Conclusions:**

Our data show that actigraphic monitoring of upper limbs can detect the laterality of the motor deficit and measure the clinical severity. These findings suggest that the above described actigraphic system could implement the existing multiparametric monitoring in stroke units.

## Background

Stroke is a disease with a high social impact causing high mortality and severe residual disability. In particular, during the acute phase it is difficult to assess the patient’s functional prognosis, especially with regard to the motor deficits that impair the activity in daily life [1, 2]. After a stroke, hemiparesis is the most common residual disability with a wide range of severity, having the upper limb the lowest functional recovery [3–5]. During the acute phase, tracking the motor performance variations of the affected upper limb versus the unaffected arm could be useful to measure clinical severity over time and to formulate a prognosis. Nowadays, the stroke unit represents the gold standard in the management of the acute stroke, since it provides a continuous multi-parametric monitoring that allows the identification of changes in cardiac functioning, blood pressure levels and hematic oxygen saturation. At the moment, the continuous monitoring of motor deficit is not implemented in the stroke unit environment. Actigraphy allows the long-term assessment of the patient’s wrist movements by means of a small solid-state sensor. Several applications of actigraphy based on accelerometers have been proposed. Indeed, actigraphy has proved its usefulness not only in sleep medicine [6], but also in other fields, for example in Parkinson tremor quantification [7]. So far, few papers have reported the use of actigraphy in stroke: these studies provided the first indication that actigraphy might be sensitive enough to detect changes in motor activity during the recovery process and to quantify motor activity in everyday life [8–14] but, no data is available about the spontaneous upper limb motor performance in the very acute phase of stroke, when the instability of clinical picture can strongly impact on prognosis and future disability and the patient needs to be monitored in an intensive care unit. Page et al. [15] have used actigraphy to evaluate rehabilitative therapies in subacute stroke subjects. Gubbi et al. [16] performed short actigraphic recordings in the hyper-acute post-stroke phase and developed an algorithm capable of calculating an index equivalent to the motor subscore of the National Institutes of Health Stroke Scale (NIHSS) that is a clinical score used to monitor changes of the neurological status during the hospital stay, with a maximum of 42 (severe stroke) and a minimum of 0 (no symptoms) [17]. The same group subsequently used that index to quantify the movement difference between arms by an intra-class correlation coefficient (ICC) analysis. They found that the greater is the difference in activity between the affected and unaffected limb, as measured by ICC, the higher is the NIHSS total score; however, they did not found any correlation between the inter-limbs motor difference and the more specific NIHSS motor sub-score [18]. Reiterer et al. actigraphically monitored motor activity of both arms in 38 patients with transient ischemic attack, ischemic lesion or non-traumatic intracerebral haemorrhage for 24 h in four different time points: 24–36 h after symptoms onset, 5–7 days later, at 3 and 6 months after symptoms onset. They demonstrated that motor performance of paretic and not paretic limbs differ during the first two time points while in the further two time points this difference was attenuated [19]. However, the actigraphic index used by the Authors did not correlate with the clinical severity in the acute phase as assessed by the NIHSS. Moreover, the Authors performed 24 h recordings in a very heterogeneous sample of patients (transient ischemic attack, ischemic lesion and non-traumatic intracerebral hemorrhage), therefore the reported data cannot be considered as representative of the ischemic stroke scenario. Urbin et al. investigated different metrics to measure upper limb motor performance in subacute and chronic ischemic and hemorrhagic stroke patients during motor training and in a free-living environment. They described the asymmetry of motor performance between paretic and non-paretic arm as a ratio between the variability of the paretic arm acceleration relative to variability of the non-paretic arm. They found that the asymmetry correlates with upper extremity function during the rehabilitative process and in a free-living environment [9, 10]. Since the authors enrolled ischemic and hemorrhagic, subacute and chronic stroke patients in an environment very different from that of a stroke unit, their results, although useful to evaluate the efficacy of different parameters, cannot be considered representative of the clinical picture of ischemic stroke patients who require intensive cares in their very acute phase.

In a previous study performed in healthy subjects, Rabuffetti et al. defined a novel numerical index to quantify upper limb motor activity and the between-limb motor asymmetry. The proposed motor activity index only depends on sensor position and not on sensor orientation (i.e. indices invariant to sensor orientation), therefore it could represent a robust approach to monitor spontaneous motor performance in complex environments such as stroke units. Moreover, the proposed asymmetry index is based on epoch-based asymmetry and not on average overall asymmetry [9, 10] therefore it could be theoretically very precise in describing motor performance over time [20]. We hypothesized that such index might effectively track the motor behavior of bed-restrained patients and could be useful to implement the multiparametric monitoring in the stroke unit environment. Therefore the aims of the present study were:

- to verify if the actigraphic asymmetry index, as calculated by Rabuffetti, can identify the paretic arm of acute stroke patients;

- to verify if such asymmetry index can properly quantify the clinical severity of acute stroke patients in the very particular environment of a stroke unit.

## Methods

### Population

Twenty consecutive middle cerebral artery stroke patients (mean age 69.2 years SD 10.1, 8 males and 12 females) were enrolled in the acute phase of stroke (3.3 ± 1.6 days after stroke onset) regardless the side, location, extension of the ischemic lesion and clinical severity. We enrolled as control group 17 patients (mean age 70.4 years SD 4.8, 7 males and 10 females) who were hospitalized for orthopedic diseases of the lower limbs, bed-restrained but not experiencing neurological symptoms. The exclusion criteria were previous ischemic stroke, hemorrhagic stroke, diagnosis of epilepsy and/or cognitive impairment, anamnestic and/or instrumental evidence of previous upper limb motor impairment. The patients were clinically evaluated by NIHSS at the beginning (NIHSS_T0_) and at the end (NIHSS_T1_) of the 24 h actigraphic recordings. When the NIHSS motor sub-score of the controlesional upper limb was scored as zero we had evaluated the presence of hand pronation as sign of clinical impairment. We coded the onset lesion load by the ASPECT score [21]. All participants were right-handed as assessed by the Edinburgh questionnaire [22]. The research was approved by the local ethics committee (Fondazione Policlinico Universitario A. Gemelli, Prot N. 0007987/17) and complies with the Helsinki Declaration. Informed written consent was obtained.

### Actigraphic recordings and data processing

The motor activity of both arms was recorded for 24 hours using two programmable actigraphic systems (EZ430-Chronos, Texas Instruments, Dallas, TX, USA) showing off as wrist-worn watches. The two devices (one for each wrist) were synchronized in order to obtain in the same time points the recordings of both limbs. The EZ430-Chronos is equipped with 4 kB RAM and 32 kB flash memory, a solid-state 3-axial sensor based on MEMS technology measuring acceleration at 33 Hz sampling rate, with a 10-bit resolution over a 4 g full scale. In real-time, the EZ430-Chronos compute the modulus of the acceleration.

The firmware, integrated in each device, segmented the acquisition in epochs of 1 minute and for each epoch calculates a motor activity index MA_e1_ (Epoch-related Motor Activity index) as the standard deviation (σ) of the acceleration module:

1 $$ M{A}_{e1}={\sigma}_{\overline{a}}.\mathrm{where}\overline{a}=\sqrt{{a_x}^2+{a_y}^2+{a_z}^2} $$

In the formula a_x_^2^, a_y_^2^, and a_z_^2^ represents the acceleration components in the three axis [20]. Additionally, a second motor index MA_e2_ is computed, in the same epochs of the first index, according to the following formula

2 $$ M{A}_{e2}=\sqrt{{\sigma_{a_x}}^2+{\sigma_{a_y}}^2+{\sigma_{a_z}}^2} $$

Where σ$\mathrm{a}$_x_^2^, σ$\mathrm{a}$_y_^2^ and σ$\mathrm{a}$_z_^2^ are the standard deviations of the acceleration components in the three axis. In this case MA_e2_ is defined as the module of the standard deviation of acceleration components.

Since gravity, a constant vector entity, determines measurable acceleration components according to the orientation of the triaxial sensor, it can be expected that a pure rotation of the wrist (or, more precisely, a pure rotation of the triaxial sensor fixed onto the wrist) modulate the measured acceleration components along the three sensor axes. According to this observation, a pure wrist rotation without a linear acceleration (for example the forearm pronation-supination typically occurring in a bed restrained patient) implies a non-zero value for the second index MA_e2_ while MA_e1_ returns a null value. On the contrary, a pure linear acceleration is reflected by non-zero values of both indices. When rotations and linear accelerations are combined, MA_e1_ is sensitive only to linear acceleration while MA_e2_ is determined by both components. However, since rotations imply a modulation of measured acceleration components up to 2 g (about 20 m/s^2^), it is obvious that MA_e2_ is much more sensitive to sensor rotations and, therefore, MA_e2_ can be assumed as a monitor specific for sensor rotations.

To describe the 24 h motor performance of each limb, we calculated the mean value of MA_e1_ and MA_e2_ (respectively MA_1_24h_ and MA_2_24h_) being the distribution of values normal. Moreover, the MA_e1_ and MA_e2_ time profiles of the right and left limbs were used to quantify the asymmetry between the motor activities of the two sides over the 24 h recordings, adopting a previously described method [20]. For both MA_e1_ and MA_e2_ indices, the synchronous values of the right and left side were scatter plotted: the values of the right wrist on x-axis and those of the left side on y-axis. Data points belonging to the quadrant bisecant indicate a strictly symmetric behavior, those in the inferior triangular area refer to epochs in which the motor activity was higher in the right side while those in the superior triangular area refer to epochs with a prevalence of left movements. The data cloud best-fitting line, passing through the axes origin and minimizing the sum of squared residuals, is the geometrical entity that summarize the asymmetry as occurred in the recordings. Such best-fitting line computationally corresponds to the first eigenvector as obtained by a singular value decomposition in a principal component analysis. Finally, after transforming, by the arctangent operator, the slope coefficient of the best-fitting line to the angle between the x-axis and the eigenvector, the percent Asymmetry Rate Index for the 24 h period (AR_24h_) was defined as follows [20]:

3 $$ A{R}_{24h}=100.0\cdot \frac{45{}^{\circ}-\alpha }{45{}^{\circ}} $$

**Fig. 1:**
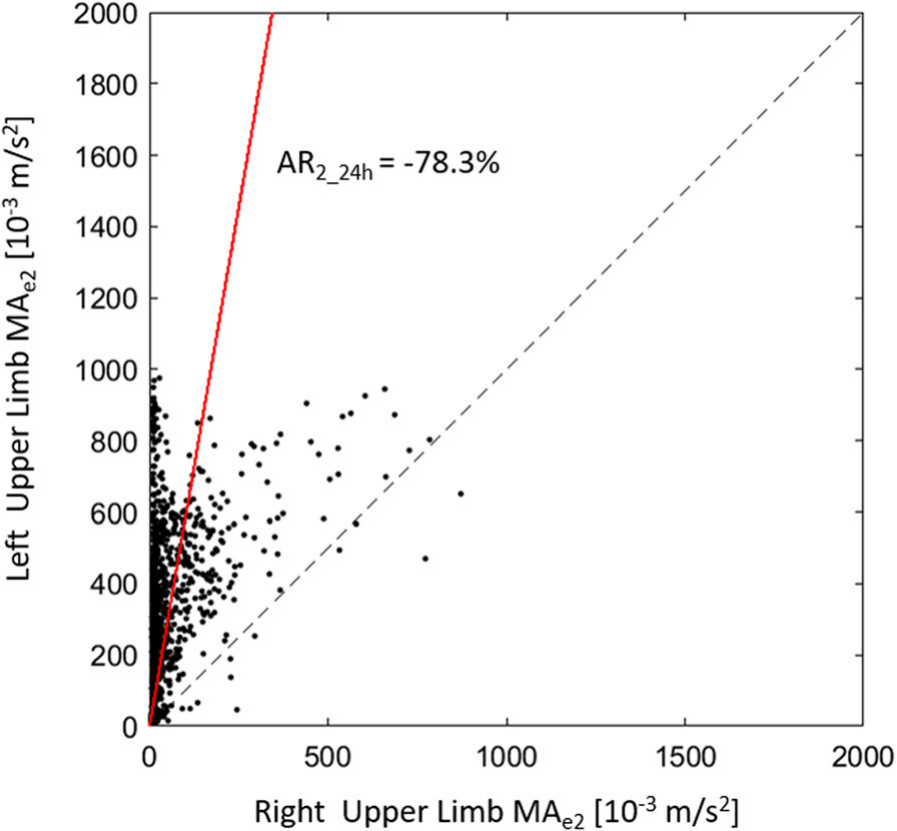
The first eigenvector identified on the cloud of 1440 data points showing a strong motor prevalence of the left arm in a right hemiplegic patient (the AR_2_24h_ index was − 78.3%)

The AR_24h_ shows a null value in a symmetric behavior, positive values for a prevalence of right side motor activity (up to a maximum of 100% if the left activity is absent) and negative values for a left side motor activity prevalence (Fig. 1). In the following we will refer to the asymmetry index of the MA_e1_ index as AR_1_24h_ and as AR_2_24h_ for MA_e2_ index. Both asymmetry indices were calculated using MATLAB (The Mathworks, Natick, USA). It is noteworthy that nurses reported on a dedicated diary every moment in which the patient was passively mobilized and the asymmetry indices (AR_1_24h_ and as AR_2_24h_) were calculated either after having removed those confounding intervals or considering them in the analysis of MA_e1_ and MA_e2_ indices.

### Statistical analysis

All statistical analyses were performed in SPSS statistics software (version 20.0). The Shapiro-Wilk probability test was used to assess the normality of the distributions. We used the Wilcoxon non-parametric test to compare the values of MA_1_24h_ and MA_2_24h_ indices between the paretic and unaffected arms and to compare NIHSS_T0_ and NIHSS_T1_ scores. We used the Mann-Whitney U Test to compare the asymmetry indices between controls and patients.

In order to evaluate the agreement between the deficit laterality measured by AR_1_24h_ and AR_2_24h_ and the clinically defined laterality, we used the Phi Coefficient. The Pearson’s test was used to correlate the degree of asymmetry between arms as measured by the absolute value of AR_1_24h_ and AR_2_24h_ with NIHSS scores (either before or after having removed the epochs with passive movements). A *p* < 0.05 was set as the level of significance.

## Results

Table 1 shows clinical, demographic and actigraphic data of the enrolled stroke sample.

**Table 1:** Demographic, Clinical and Actigraphic Characteristics of the Patients

Patient	Age (years)	Gender	Hemiparetic Side	NIHSS_T0_ total score	NIHSS_T1_ total score	NIHSS_T0_ motor sub-score	NIHSS_T1_ motor sub-score	ASPECT	Days between stroke onset and registration	AR_1_24h_	AR_2_24h_
01	58	M	L	14	14	4	4	10	4	5.3%	25.8%
02	87	F	R	2	2	0	0	10	3	3.4%	− 31.5%
03	57	F	R	1	1	0	0	8	4	−24.7%	−19.7%
04	77	F	R	2	2	0	0	10	3	−17.7%	− 13.1%
05	62	M	R	11	11	4	4	7	1	− 65.6%	− 78.3%
06	66	F	R	7	7	4	4	10	5	−70.5%	− 83.2%
07	70	F	L	6	6	3	3	10	3	10.6%	32.6%
08	65	M	L	10	10	4	4	7	2	51.6%	92.1%
09	84	M	L	11	11	3	3	10	3	51.9%	82.4%
10	84	F	L	16	16	4	4	10	1	68.7%	79.8%
11	60	F	R	1	1	0	0	10	2	−17.8%	−16.1%
12	59	M	R	1	1	0	0	10	5	3.6%	− 11.4%
13	78	F	L	8	8	3	3	10	6	68.3%	84.9%
14	54	M	L	4	4	2	2	8	1	−2.2%	11.8%
15	60	F	L	10	10	4	4	10	5	4.6%	77.2%
16	74	F	R	18	18	4	4	10	6	−43.0%	−79.5%
17	63	F	L	15	15	4	4	5	1	51.5%	77.6%
18	78	M	L	11	11	4	4	8	3	6.9%	77.0%
19	75	M	R	2	2	0	0	10	4	−4.3%	11.1%
20	73	F	L	8	7	4	3	6	4	67.8%	86.2%
Summary row (mean ± SD or range)	69.2 ± 10.1	8 M 12 F	9 right 11 left	7.9 ± 5.4	7.9 ± 5.4	2.6 ± 1.8	2.5 ± 1.8	9 ± 1.6	3.3 ± 1.6	[−70.5 68.7]%	[−83.2 92.1]%

L = left, R = right, AR_1_24h_ = Asymmetry Rate Index of the MA_e1_ index for the 24 h period, AR_2_24h_ = Asymmetry Rate Index of the MA_e2_ index for the 24 h period, NIHSS_T0_/NIHSS_T0_ motor sub-score = at the beginning of the 24 h actigraphic recordings, NIHSS_T1_/NIHSS_T1_ motor sub-score = at the end of the 24 h actigraphic recordings.

Table 2 shows clinical, demographic and actigraphic data of the enrolled control sample.

**Table 2:** Demographic, Clinical and Actigraphic Characteristics of the Controls

Control	Age (years)	Gender	Orthopedic intervention	AR_1_24h_	AR_2_24h_
01	65	M	hip replacement	− 26.3%	−1.2%
02	72	F	knee replacement	24.8%	20.2%
03	68	F	knee replacement	−19.7%	−13.4%
04	77	F	hip replacement	31.9%	10.9%
05	68	M	hip replacement	−0.8%	−1.7%
06	71	F	knee replacement	44.6%	−10.8%
07	69	F	hip replacement	−0.1%	−14.0%
08	62	M	hip replacement	−22.1%	0.1%
09	71	M	hip replacement	−39.2%	−21.1%
10	75	F	hip replacement	4.4%	−0.4%
11	60	F	knee replacement	3.1%	4.6%
12	75	M	hip replacement	1.7%	−16.5%
13	75	F	hip replacement	2.6%	−10.8%
14	70	M	knee replacement	20.3%	18.8%
15	74	F	hip replacement	30.5%	8.3%
16	74	M	hip replacement	−6.8%	−3.9%
17	71	F	knee replacement	2.6%	11.3%
Summary row (mean ± SD or range)	70.4 ± 4.8	7 M 10 F	11 hip replacement 6 knee replacement	[−39.2 44.6]%	[−21.1 20.2]%

AR_1_24h_ = Asymmetry Rate Index of the MA_e1_ index for the 24 h period, AR_2_24h_ = Asymmetry Rate Index of the MA_e2_ index for the 24 h period.

**Fig. 2:**
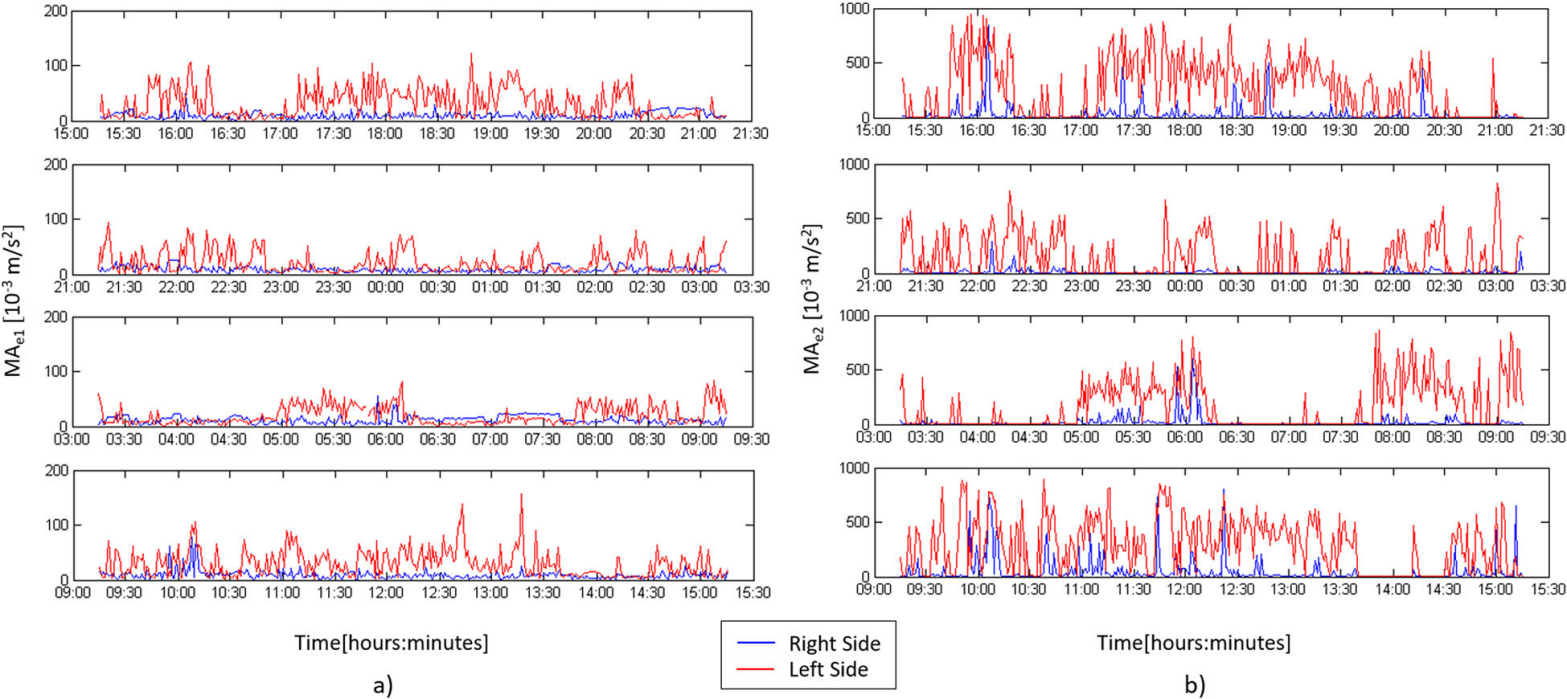
MA_e1_ (**a**) and MA_e2_ (**b**) profiles during the 24 h actigraphyc monitoring in a paradigmatic patient with right hemiplegic side: the blue line refers to the movement of the right wrist while the red profile to the left wrist. The figure illustrates the MA_e1_ and MA_e2_ profiles without excluding the recordings corresponding to passive movements due to nurses’ activities

**Fig. 3:**
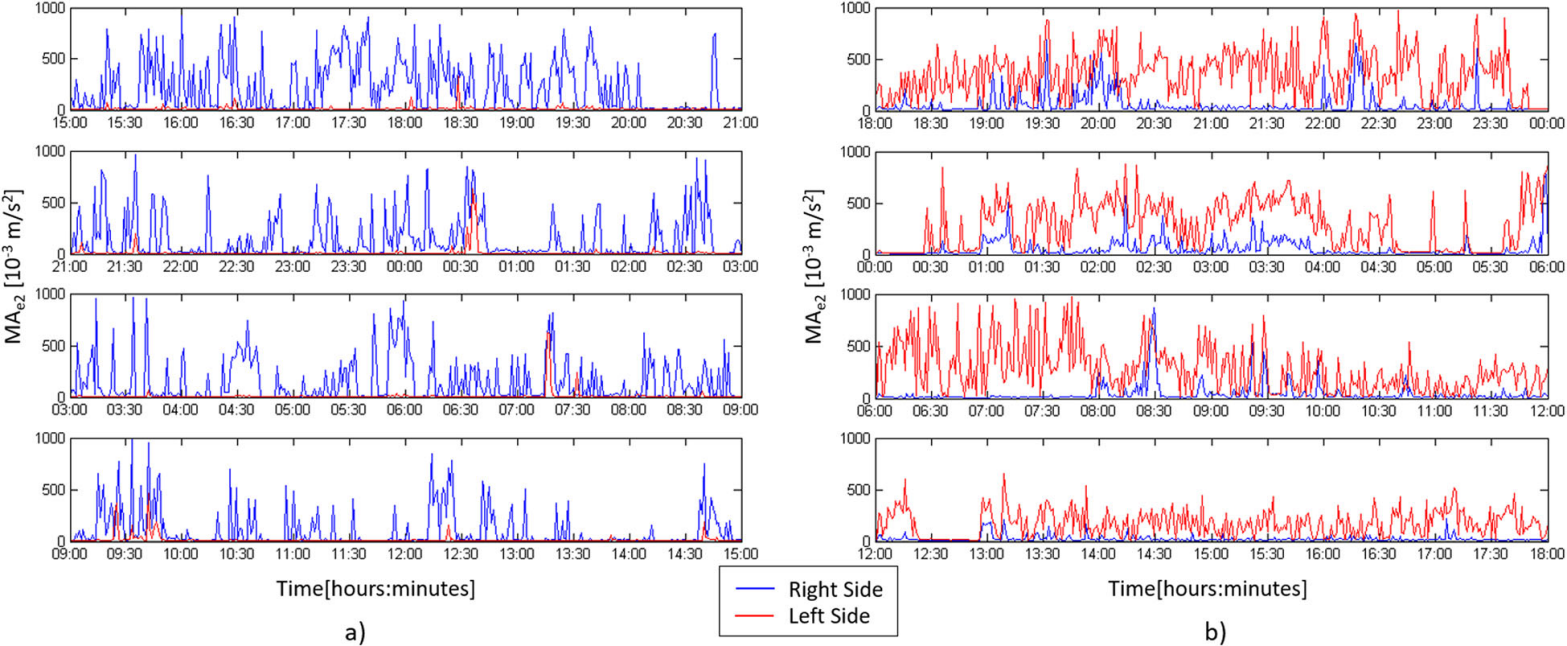
MA_e2_ profiles of two patients respectively with left (**a**) and right (**b**) hemiplegia. The blue line refers to the movement of the right wrist while the red profile to the one of the left wrist

**Fig. 4:**
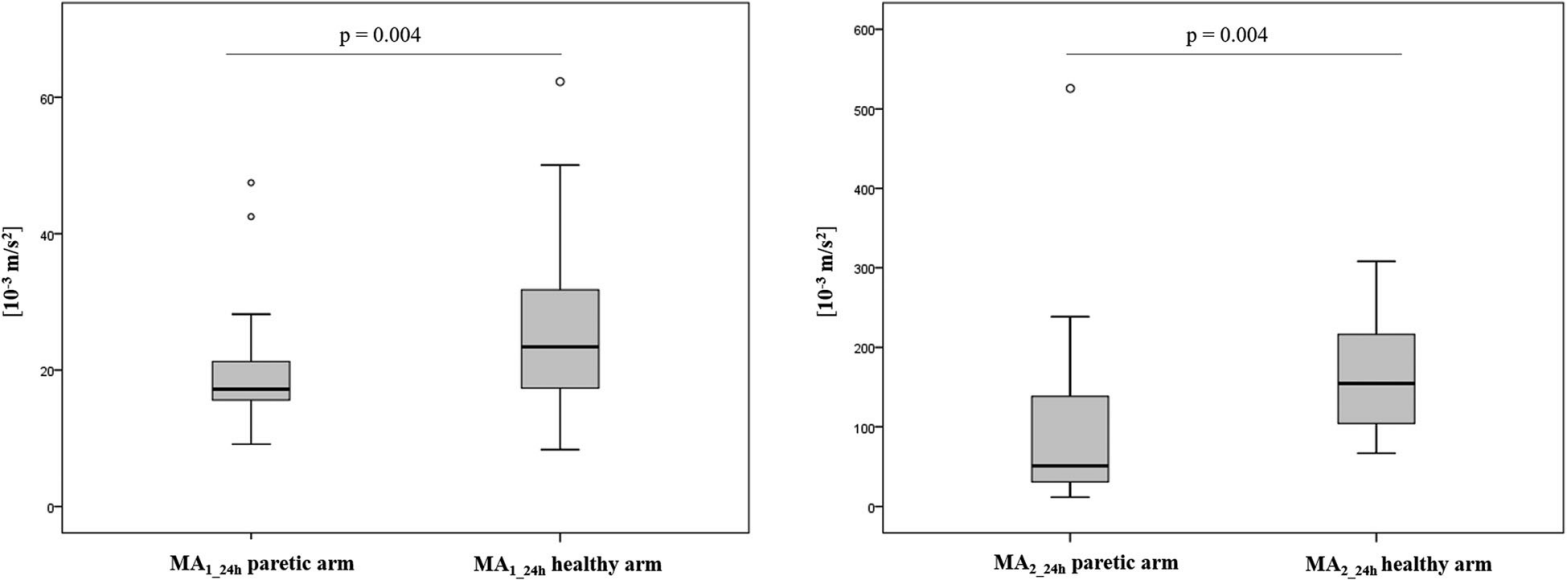
MA_1_24h_ and MA_2_24h_ indices: comparison between the paretic and unaffected arm. Both indices are smaller in the paretic than in unaffected arm

**Fig. 5:**
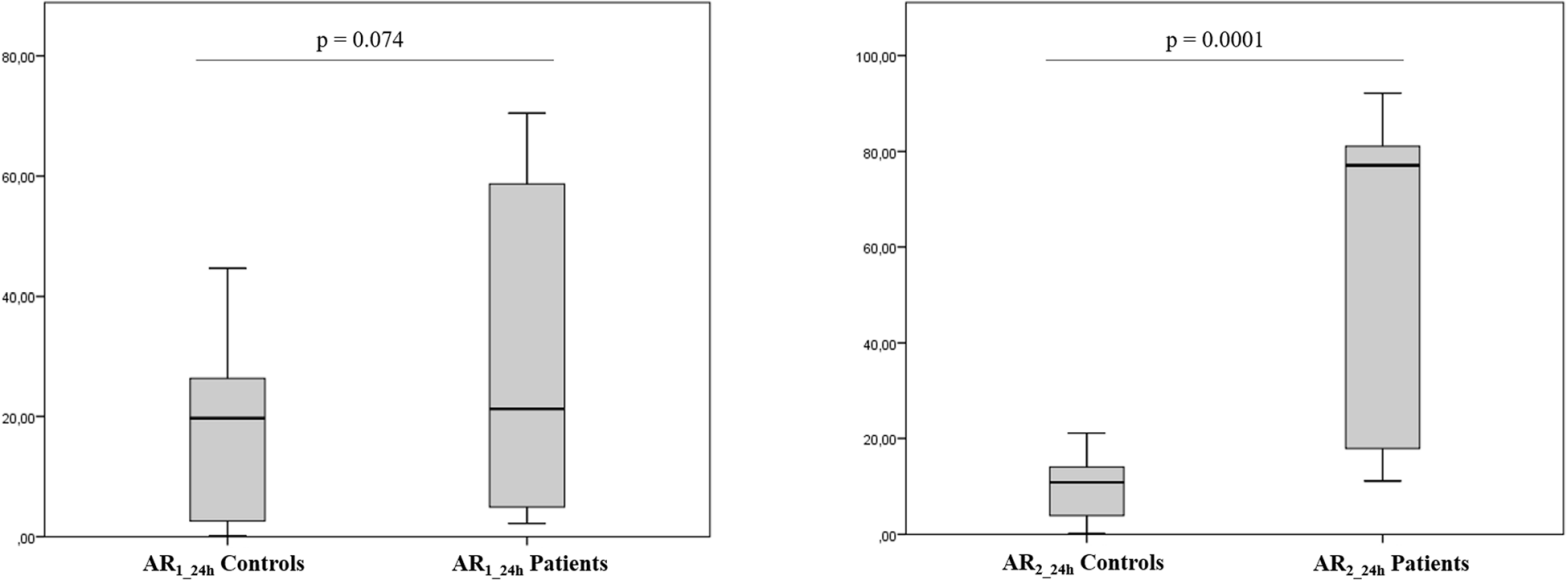
AR_1_24_ and AR_2_24_ indices: comparison between patients and controls

Figure 2 illustrates MA_e1_ and MA_e2_ profiles during the 24 h actigraphic monitoring in a paradigmatic patient with right hemiplegia: the blue line refers to the movement of the right wrist while the red profile to the left wrist. MA_e1_ shows very low values during the whole recording (Fig. 2a); on the opposite MA_e2_ shows greater values (Fig. 2b). Figure 3 shows the MA_e2_ profiles of two patients respectively with left (Fig. 3a) and right hemiplegia (Fig. 3b). MA_e2_ profiles show a clear prevalence of the right limb movement in the left hemiplegic patient (AR_2_24h_ = + 92.1%) (Fig. 3a) and vice versa a prevalence of the left movement in the patient with right hemiplegia (AR_2_24h_ = − 78.3%) (Fig. 3b). We found no difference between NIHSS_T0_ and NIHSS_T1_ scores. Both MA_1_24h_ and MA_2_24h_ indices were smaller in the paretic than in the unaffected arm (respectively *p* = 0.004 and *p* = 0.004) (Fig. 4). We found that AR_2_24h_ was greater in stroke patients than in controls, while AR_1_24h_ did not differ between groups (Fig. 5). AR_2_24h_ showed a better capability (95% of paretic arms correctly identified, Phi Coefficient: 0.903) to discriminate the laterality of the clinical deficit than AR_1_24h_ (85% of paretic arms correctly identified, Phi Coefficient: 0.698) (Table 3).

**Table 3:** Deficit laterality as described by AR_1___24h_ and AR_2_24h_

	Hemiparetic Side as assessed by AR_1_24h_			Hemiparetic Side as assessed by AR_2_24h_		
	Right	Left	Total	Right	Left	Total
Right Hemiparesis	7	2	9	8	1	9
Left Hemiparesis	1	10	11	0	11	11
Total	8	12	20	8	12	20

AR_1_24h_ = Asymmetry Rate Index of the MA_e_ index for the 24 h period,

AR_2_24h_ = Asymmetry Rate Index of the MA_e2_ index for the 24 h period.

**Fig. 6:**
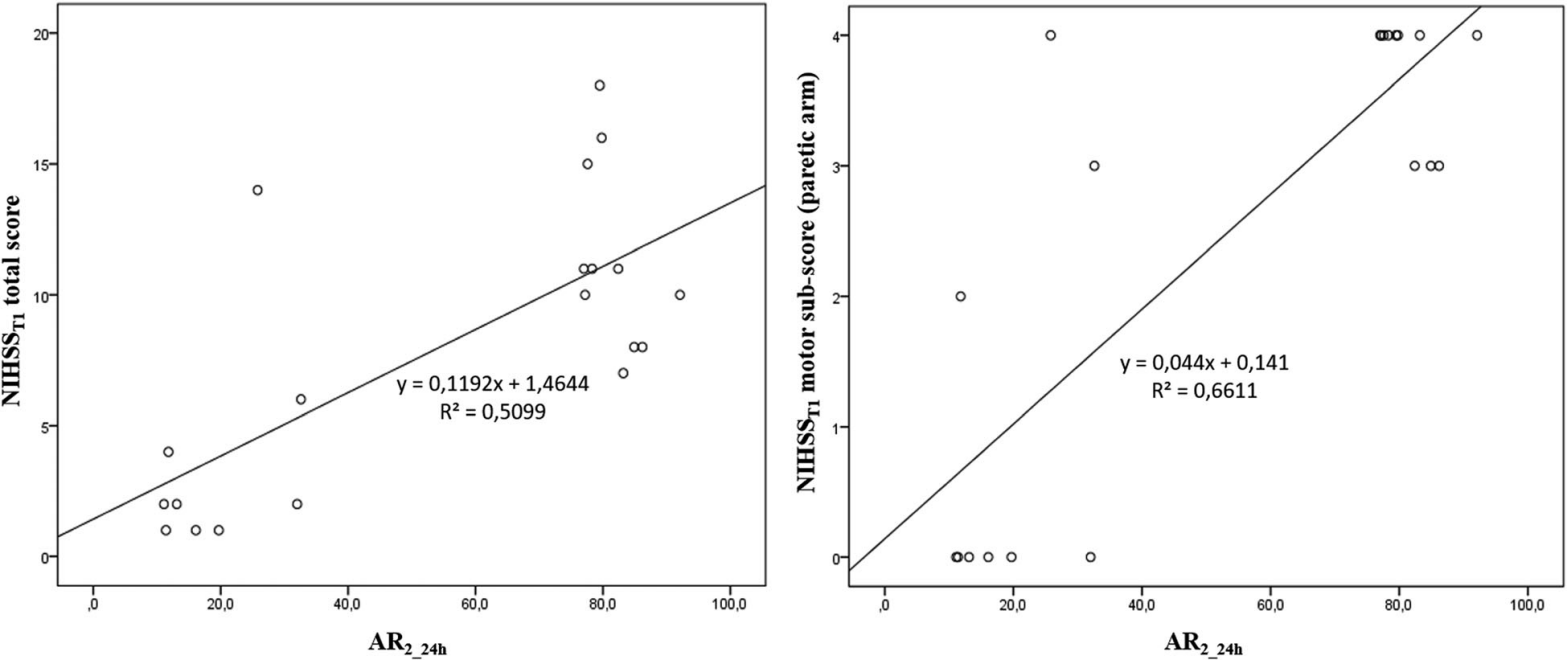
Correlation between AR_2_24h_ and NIHSS_T1_ total scores and between AR_2_24h_ and NIHSS_T1_ sub-score relative to the paretic upper limb

Moreover, we found a positive correlation between AR_2_24h_ and NIHSS total scores (r: 0.714, *p* < 0.001 for NIHSS_T1_, CI95%: 0.42–0.90) and between AR_2_24h_ and the sub-score relative to the paretic upper limb (r: 0.812, *p* < 0.001 for T1 sub score, CI95%: 0.62–0.96) (Fig. 6). The correlation between AR_2_24h_ and the NIHSS scores worse when the epochs related to passive movements were not rejected (correlation between AR_2_24h_ and NIHSS_T1_ was r: 0.408, *p*: 0.074, CI95%: 0.03–0.73; between AR_2_24h_ and NIHSS sub-score relative to the paretic upper limb at T1 r: 0.546, *p*: 0.01, CI95%: 0.23–0.79).

## Discussion

Our data show that actigraphic monitoring of upper limbs spontaneous movements is able to distinguish the paretic arm from the healthy one. We have tested two different indices, namely MA_e1_ and MA_e2_, documenting a statistically greater motor activity in the healthy side. However, when the asymmetry indices AR_1_24h_ and AR_2_24h_ (respectively based on MA_e1_ and MA_e2_) were used to describe the motor performance of the affected arm relatively to the performance of the unaffected one, the two asymmetry indices present different patterns. On the one hand, the AR_2_24h_ shows a greater asymmetry in stroke patients than in the control group, it is informative about the severity of the neurological symptoms (strong correlation with the NIHSS upper-limb score and with the NIHSS total score) and predictive of the laterality of the clinical impairment. On the other hand, AR_1_24h_ does not allow to distinguish stroke patients from bed-restrained subjects without motor impairment of upper limbs. Indeed AR_1_24h_ does not differ between stroke patients and control group. Technically, the main difference between AR_1_24h_ and AR_2_24h_ consists in the different way in which MA_e1_ and MA_e2_ are computed, being MA_e2_ based on the evaluation of each component of the acceleration and MA_e1_ on the variation of the module of acceleration [20]. This element is pivotal in determining a higher capability of AR_2_24h_ to describe the motor performance in our sample of patients who may experience a severe limitation of the movement: the MA_e2_ definition, contrarily to the MA_e1_ one, is able to detect sensor rotations, i.e. forearm pronosupination in the considered bedridden patients [20]. It is noteworthy that the AR_2_24h_ was able to identify a right slight motor deficit (AR_2_24h_ ranges from − 11.4% to − 31.5%) even in five out of the six patients with left hemispheric stroke and whose clinical scores failed to show a motor deficit (the five patients presented hand pronation but a NIHSS arm subscore = 0 meaning absence of arm drift). In one patient with left hemispheric stroke and NIHSS arm subscore = 0, AR_2_24h_ shows a slight motor prevalence of the right arm (patient 19 in Table 1, AR_2_24h_: + 11.1%) revealing no right motor deficit: this apparently contradictory result is simply explained by the absence of a motor deficit of the right upper limb (no hand pronation) and the presence of a physiological motor prevalence of the dominant (right) side, as observed in healthy subjects [23, 24].

Gebruers and colleagues performed a study similar to ours in acute stroke patients and highlighted that actigraphic measurements show moderate correlation with NIHSS total score [25] and can contribute to predict clinical recovery as measured by Fugl-Meyer and modified Rankin scale [26, 27] More deeply, the actigraphic recordings of the paretic arm correlated better with NIHSS total score than the ratio between the activities of the two arms. Moreover they found that the NIHSS sub-score relative to the paretic upper limb was related with actigraphic variables only in patients with left hemispheric lesions. The moderate correlation between the actigraphic recordings and the clinical picture is probably explained by the method adopted to analyze the actigraphic findings. The authors used the Proportional Integrating Measure (PIM), a modality of movement measurement consisting in integrating the signal from the sensor to calculate the area under the rectified curve. This area was calculated for the affected and the unaffected upper arms and presented as an amount of activity. Ratio was calculated by dividing the area under the rectified curve of the affected upper limb by the area under the curve of the unaffected side. Therefore the parameters described represent an overall evaluation of movement over the 24 h without considering the time profile of changes of motor activity. In our approach we calculated the asymmetry index not as a simple ratio between the overall motor activities of the two sides but we considered the time profiles of MA_e1_ and MA_e2_ in both arms to identify the eigenvectors which represent the data cloud best-fitting line. Then the asymmetry indices were computed by measuring the angle between the x-axes and the eigenvectors. In this way the asymmetry indices describe the asymmetry between synchronous values related to the movement of the two limbs and independently by knowing which was the paretic side. It is remarkable that we did not select our patients according to the clinical picture and therefore we also enrolled patients scored as zero to the NIHSS upper limb sub-score. This choice could reduce the capability of asymmetry indices to discriminate between affected and unaffected limb but our results demonstrate that the AR_2_24h_ index has an excellent capability to discriminate the laterality of the clinical deficit and a very good correlation with NIHSS total score and the sub-score relative to the paretic side [25]. Our approach in measuring motor asymmetry by the MA_e1_index was conceptually similar but not equal to that adopted by Urbin and colleagues in a sample of subacute/chronic ischemic and hemorrhagic stroke patients during the rehabilitation process or in an everyday free-living environment [9, 10]. From an engineering perspective, a first difference consists in calculating MA_e1_ index without removing epochs where no acceleration occurred. In fact, in our environment (stroke unit with acute patients monitored at bed) no acceleration could indicate plegia of the arm therefore, removing those epochs would have meant removing a clinical crucial information. Urbin and colleagues made a different choice because their objective was to characterize how and not how often movement occurs after rehabilitation training or in everyday free-living environment. We needed to both characterize the quality of the spontaneous movement and measuring the amount/frequency of movement which is pivotal in a stroke unit. Moreover the asymmetry calculation is different because the variation ratio [9, 10], being a ratio between standard deviations of the mean accelerations of the arms, describes the asymmetry during the overall recording period without considering the time profile of changes of motor activity, as the eigenvector calculation does. Since upper limb movements have a linear and rotational component of acceleration, we have also evaluated the MA_e2_ index which is sensitive to both components although it is much more sensitive to sensor rotations. Our approach was to evaluate both indices in order to verify if both were necessary to properly describe the clinical picture or if one of them was sufficient. From a clinical perspective, Urbin and colleagues evaluated a composite sample of hemorrhagic and ischemic patients in the sub-acute/chronic phase and in an environment different than the intensive care unit, therefore their results cannot directly be transferred to our clinical context.

As expected, after having removed from the actigraphic recordings the epochs relative to the moments when patients were passively mobilized, the correlation between asymmetry indices and the clinical picture as measured by NIHSS scores improves .

## Conclusions

The results of our feasibility study demonstrate that, in the acute phase of ischemic stroke, the asymmetry between upper limbs measured by the actigraphic AR_2_24h_ index correlates with the overall neurological clinical status and with the paretic upper limb motor deficit as measured by NIHSS. Moreover, the AR_2_24h_ index has a very good capability to identify the paretic arm. These findings suggest that the above described index could implement the existing multiparametric monitoring in stroke unit. In this view, a further technological advance, with improvement of energetic efficiency of the accelerometers, could allow a long-lasting recording from the onset of the symptoms to the discharge from the stroke unit, providing a real time evaluation of motor symptoms during the unstable period of the stroke acute phase.

## Data Availability

The dataset used and/or analyzed during the current study available from the corresponding author on reasonable request.

## Abbreviations

AR_24h_: Asymmetry Rate Index
CI: Confidence Intervals
ICC: intra-class correlation coefficient
MA_e1_: Epoch-related Motor Activity index
MA_e2_: Epoch-related Motor Activity index 2
NIHSS: National Institutes of Health Stroke Scale
PIM: Proportional Integrating Measure
